# Photobiomodulation Therapy at 660 nm Inhibits Hippocampal Neuroinflammation in a Lipopolysaccharide-Challenged Rat Model

**DOI:** 10.3390/biomedicines12112514

**Published:** 2024-11-03

**Authors:** Tae-Mi Jung, Jong-Ha Lee, Jin-Chul Heo, Chang-Hyun Kim

**Affiliations:** 1 Department of Computer Engineering, Keimyung University, Daegu 42601, Republic of Korea; 1114436@stu.kmu.ac.kr (T.-M.J.); washingbuffer@ms.kmu.ac.kr (J.-C.H.); 2 Department of Neurosurgery, Keimyung University Dongsan Hospital, Daegu 42601, Republic of Korea

**Keywords:** photobiomodulation therapy, low-level light therapy, hippocampus, brain inflammation

## Abstract

Background/Objectives: Neuroinflammation is associated with the progression of various brain diseases, and the management of neuroinflammation-induced neural damage is a crucial aspect of treating neurological disorders. This study investigated the anti-inflammatory efficacy of photobiomodulation therapy (PBMT) using 660 nm phototherapy in a rat model with lipopolysaccharide (LPS)-induced neuroinflammation. Methods: We induced inflammation in rat brains via intraperitoneal injection of LPS and subjected the treatment group to 660 nm phototherapy to examine its protective effect against hippocampal damage based on pathological, histological, and immunohistochemical tissue analyses. Results: The 660 nm treated rats showed a significant decrease in hippocampal structural damage and cell death compared to the LPS-treated group. We observed reduced expression of the inflammation markers GFAP, TNF-α, and IL-1β in the hippocampus of the treatment group, and an increase in SIRT1 expression across all hippocampal regions. Conclusions: This study presents a promising method for controlling neuroinflammation and providing neuroprotection and inflammation relief. PBMT represents a non-invasive therapeutic approach with minimal side effects ensured through the proper control of light irradiation.

## 1. Introduction

Neuroinflammation is an important driver of cognitive disorders and neurodegenerative diseases such as Alzheimer’s disease (AD), Parkinson’s disease (PD), Huntington’s disease, multiple sclerosis (MS), and amyotrophic lateral sclerosis (ALS) [1,2,3]. Microglia, the brain’s macrophages, play a crucial role in the onset and development of neuroinflammation [4]. While acute neuroinflammation serves a protective role in the body, chronic neuroinflammation can damage neural tissue [5]. Whether neuroinflammation has beneficial or detrimental effects on the brain largely depends on the duration of the inflammatory response and type of microglial activation [6]. Microglia remove metabolic waste and toxins and [7], upon stimulation, they migrate to lesions to clear damaged cells [8]. Overactive or persistent activation of microglia leads to neurodegeneration and increased inflammatory cytokine production, damaging brain tissue [9]. Activation of microglia leads to the release of inflammatory cytokines such as nitric oxide (NO), tumor necrosis factor (TNF)-α, and interleukin (IL)-1β, characteristic markers of AD, PD, MS, and cerebral ischemia [6,10,11].

Lipopolysaccharide (LPS) expressed in the microglia of the central nervous system activates these cells to produce inflammatory cytokines [10,12]. These cytokines are key mediators of neuroinflammation, and their overactivation can induce cognitive impairment and depression in animals [13,14]. Given its clinical relevance, LPS administration is commonly used for studying neuroinflammation-related diseases in mice [10].

Photobiomodulation therapy (PBMT), also known as low-level laser therapy, is widely used to treat various medical conditions including pain, inflammation, blood disorders, musculoskeletal disorders, and tissue regeneration [15]. Recent studies have expanded its application to brain disorders, where it enhanced brain blood flow, metabolic activity, neurogenesis, and neuroprotection, particularly in central nervous system-related disorders [16]. PBMT exerts its potent beneficial effects through the activation of antioxidative and anti-inflammatory pathways [16,17,18]. It also enhances mitochondrial function, which is crucial in promoting cellular energy metabolism and supporting cell survival [19,20]. Light energy absorbed by mitochondria increases ATP production and decreases reactive oxygen species (ROS) levels, thus preventing local tissue damage [21]. PBMT has also been reported to reduce neuroinflammation and enhance the brain’s antioxidant capabilities, improving neuronal survival and function [18,22,23]. The 660 nm wavelength has been shown to induce brain-derived neurotrophic factor (BDNF) activity in hippocampal tissue, thereby alleviating oxidative stress and cellular damage [24], alleviating pain, improving ability to move, and reducing fibroblast invasion [25], and is effective for treating spinal cord injury [26].

We previously confirmed that light irradiation at 660 nm inhibited hippocampal damage induced by oxidative stress and increased BDNF expression, thereby reducing neuronal apoptosis [24]. This study aimed to verify the effects of 660 nm wavelength phototherapy on LPS-induced neuroinflammation in rat brains, especially regarding the hippocampal tissue.

## 2. Materials and Methods

### 2.1. Materials and Reagents

We used sterile-filtered LPS (1 mg/mL in saline, Sigma-Aldrich, St. Louis, MO, USA) in our experiment. GFAP (ab68428), TNF-α (ab1793), IL-1β (ab283822), and SIRT1 (ab110304) antibodies for immunohistochemistry were obtained from Abcam (Toronto, ON, Canada). Then the data was analyzed using a Wavelength Dispersive X-Ray Fluorescence Spectrometer (XRF-1800, Shimadzu, Kyoto, Japan) at Intelligent Construction System Core-Support Center, Keimyung University, Republic of Korea.

### 2.2. Animal Care and Ethical Considerations

All procedures were performed in compliance with the Guiding Principles in the Care and Use of Animals (National Research Council, 1996) and in-house guidelines of Keimyung University (29), and approved by the Animal Experiment Ethics Committee of Keimyung University (KM-2022-01R1). Briefly, 8-week-old male Sprague–Dawley (SD) rats (250–300 g) were purchased from Hyochang Science (Daegu, Republic of Korea) and provided with a commercial diet and water ad libitum. The animals were housed under a 12 h light/dark cycle at 22 ± 1 °C and a humidity of 50 ± 5%. Animals were allowed to acclimate to the laboratory environment for at least 1 week prior to the experiments.

### 2.3. LPS-Induced Neuroinflammation Model and Immunohistochemistry

**Figure 1:**
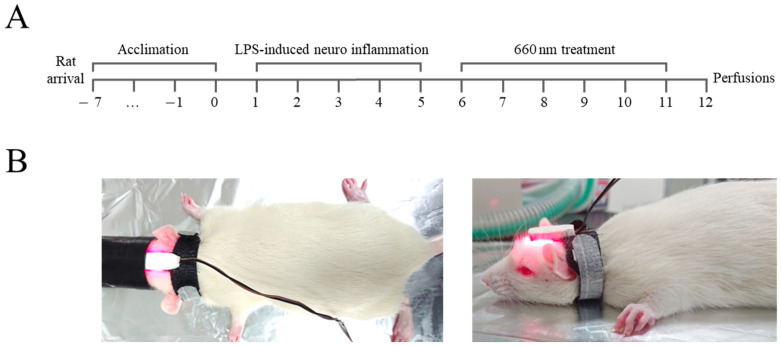
Experimental schedule (**A**) for external PBMT in a rat model (**B**).

Rats were treated intraperitoneally with LPS (250 μg/kg) or saline (0.9% NaCl) once per day for 5 d and divided into three groups (*n* = 5/group): (1) control (CON, vehicle-treated), (2) LPS (LPS-induced neuroinflammation model), and (3) LPS with 660 nm (660 nm light irradiation, 30 min/d for 6 d) [10]. A 660 nm wavelength LED light source was used for brain stimulation. The device was attached to the head of the rat and designed to irradiate for a set period of time (Figure 1, Table 1). The hippocampus was fixed using 10% paraformaldehyde in 0.1 M PBS (pH 7.4) and embedded in paraffin. Tissue sections were stained with hematoxylin–eosin (H&E) to evaluate overall tissue morphology and then subjected to immunohistochemical analysis. Paraffin blocks were sliced into 4–6 μm sections, which were then mounted on glass slides. The sections were deparaffinized using xylene and a series of ethanol dilutions, stained with H&E, and then immunostained to label cells that had migrated into the hippocampus. To quench endogenous peroxidase activity, all slides were incubated overnight at room temperature in 0.3% H_2_O_2_ in methanol. Primary antibodies, diluted at 1:500 to 1:2000 (GFAP (1:500), TNF-α (1:2000), IL-1β (1:5000), and SIRT1 (1:2000)) in PBS with 1% bovine serum albumin (BSA), were applied overnight at 4 °C. The slides were then incubated with HRP-conjugated secondary antibodies (diluted 1:500 in 5% BSA in PBS) at 37 °C for 1 h. Additionally, slides were stained with 1% Schiff’s reagent and Mayer’s hematoxylin for 5 min at room temperature. The proportion of immunostained positive cells in the tissue was compared to control results after three repeated experiments. After the scheduled treatment, the brain was excised, tissues sections were stained with hematoxylin and eosin (H&E), and pathological analysis was performed using a slide scanner (MoticEasyScan One, Motic, BC, CAN) and quantified using ImageJ software (ver. 1.54g, National Institutes of Health, Bethesda, MD, USA). After converting the image type to 8 bit in the ImageJ program, the dyed area was detected using the threshold value. Afterwards, the analysis values were obtained and statistically processed.

### 2.4. Statistical Analysis

Data are expressed as the mean ± standard deviation. Differences between the groups were determined using Student’s *t*-test for independent means in Microsoft Excel (Microsoft, Redmond, WA, USA). Statistical significance was set at *p* < 0.05 and 0.01.

## 3. Results

### 3.1. Phototherapy Inhibits LPS-Induced Hippocampal Cellular Damage

**Figure 2:**
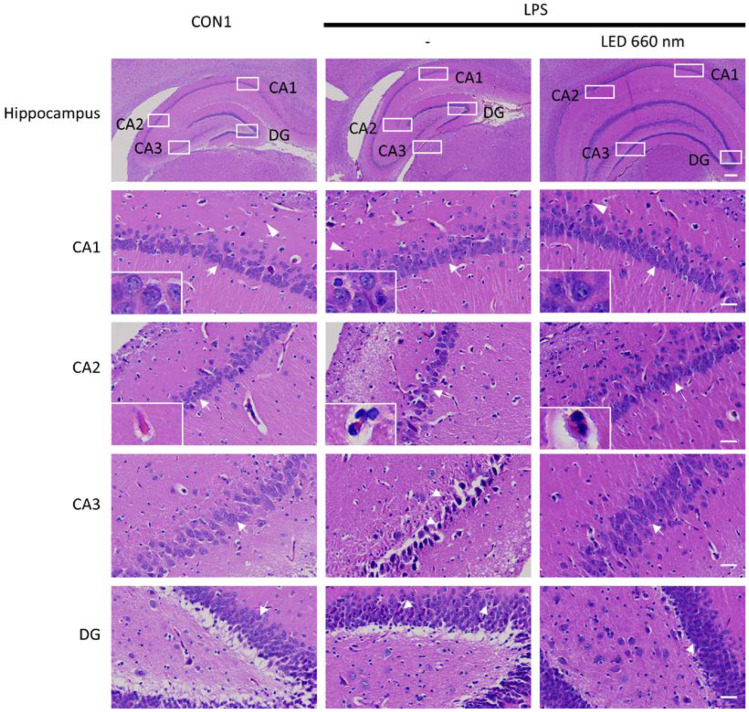
Light irradiation at 660 nm inhibits LPS-induced neuroinflammation in the hippocampus. Representative images of H&E-stained sections from the CA1, CA2, CA3, and DG regions after inducing inflammation using LPS. The normal/control group had neurons with a typical pyramidal shape and well-developed dendrites (arrows). Neurons in the LPS-administered group had expanded neuronal cell bodies and lost the dendrites. Glial cells showed a darkly stained distorted shape, vacuoles were observed around the cells (arrowheads), and immune cells in Virchow–Robin spaces (high-magnification image). Scale bars = 300 µm and 30 µm.

Morphological changes in the pyramidal cells of hippocampal areas CA1, CA2, and CA3 were measured through H&E staining. In the normal group, the hippocampal areas displayed a clear pyramidal cell layer with no lymphocyte infiltration and distinct fibrous structures. In LPS-treated rats, there was a significant reduction in pyramidal cell numbers, with disordered cell arrangement and unclear layers due to substantial lymphocyte infiltration. Pyramidal cells were notably damaged, with condensed nuclei. The fibrous structure was disordered and unclear, with vacuolation observed in the cytoplasm. These results indicated that LPS induced morphological changes in the hippocampal pyramidal cells, suggesting inflammation (Figure 2).

In the LPS-treated group, the pyramidal cell layers in CA1, CA2, and CA3 appeared atrophied. Pyramidal cells were small with condensed nuclei, dark cytoplasm, and disordered morphology. Some cells had lost their pyramidal shape, and some surrounding cells showed atrophic forms. In the polymorphic layer, there was an increase in glial cells (astrocytes). The DG area lost some cells and had greatly disordered cell layers. The granular layer was atrophied with degenerated granule cells showing vacuolation and dark condensed nuclei.

After phototherapy, the neuroinflammation-related histological changes were less noticeable than following inflammation induction. The pyramidal layers in CA1 and CA3 displayed relatively normal thicknesses. The pyramidal cells in the CA region appeared preserved with vesicular nuclei and pale basophilic cytoplasm. Some cells with condensed nuclei were also observed, though vacuolation due to cell atrophy was significantly reduced. The histological structure of the DG was mostly preserved, with densely arranged granule cells with round pale vesicular nuclei.

These findings indicated that the LPS treatment effectively induced an inflammatory response in all areas of the hippocampus, damaging neural structures, whereas exposure to 660 nm phototherapy mitigated these inflammatory responses and associated neural damage.

### 3.2. Phototherapy Inhibits the LPS-Induced Hippocampal Inflammatory Response

**Figure 3:**
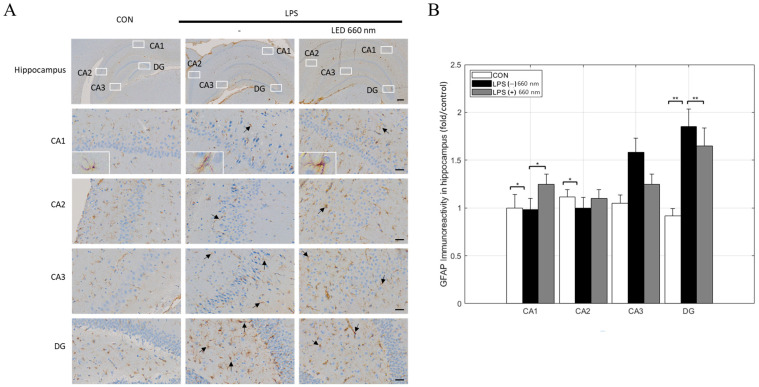
Light irradiation at 660 nm inhibits LPS-induced GFAP expression in the hippocampus. (**A**) Representative images of immunohistochemical staining for GFAP expression in the hippocampus (arrows). (**B**) GFAP-positive cell numbers in the CA1, CA2, CA3, and DG regions (scale bars = 300 µm and 30 µm; * *p* < 0.05, ** *p* < 0.001).

GFAP is commonly upregulated in astrocytes in response to central nervous system damage and is widely used as a marker of astrocyte reactivity. In this study, there was no significant difference in GFAP expression across hippocampal areas (CA1, CA2, CA3, DG) in the CON group. In the LPS-treated group, GFAP expression increased significantly in the CA3 and DG areas, suggesting that astrocytes were activated by the LPS-induced inflammatory response. However, after treatment with 660 nm wavelength LED light, GFAP expression decreased in the CA3 and DG areas (Figure 3A,B).

**Figure 4:**
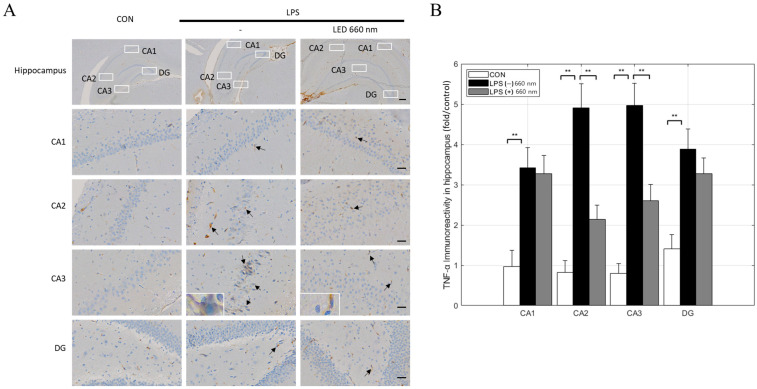
Light irradiation at 660 nm inhibits LPS-induced TNF-α expression in the hippocampus. (**A**) Representative images of immunohistochemical staining for TNF-α in the hippocampus (arrows). (**B**) TNF-α-positive cell numbers in the CA1, CA2, CA3, and DG regions (scale bars = 300 µm and 30 µm; ** *p* < 0.001).

TNF-α is a key inflammatory regulator of brain inflammation. In a healthy brain, TNF-α is expressed at very low levels in various brain cells, including neurons. However, during inflammation, microglia and astrocytes express and release high levels of TNF-α. Compared to the CON group, the number of TNF-α-positive cells was significantly higher in all hippocampal areas in the LPS-treated group. In contrast, the 660 nm treated group showed a significant reduction in the number of TNF-α-positive cells, specifically in the CA2 and CA3 areas (Figure 4A,B).

**Figure 5:**
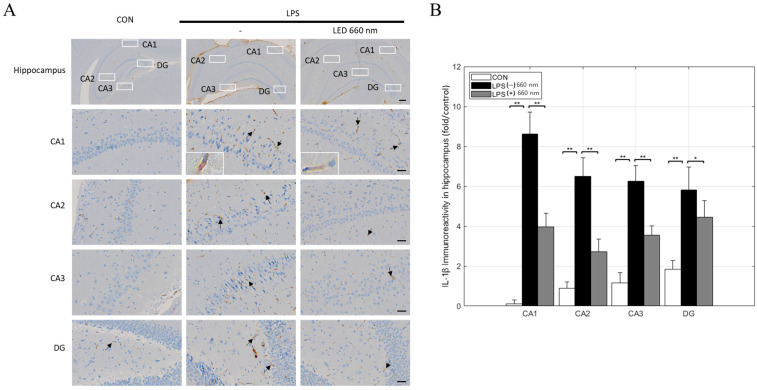
Light irradiation at 660 nm inhibits LPS-induced IL-1β expression in the hippocampus. (**A**) Representative images of immunohistochemical staining for IL-1β in the hippocampus (arrows). (**B**) IL-1β-positive cell numbers in the CA1, CA2, CA3, and DG regions (scale bars = 300 µm and 30 µm; * *p* < 0.05, ** *p* < 0.001).

IL-1β maintains the immune response in central nervous system diseases and activates microglia and astrocytes. Compared to the CON group, IL-1β expression increased significantly in the hippocampus of the inflammation-induced group. After treatment with 660 nm, IL-1β expression decreased across all hippocampal areas (Figure 5A,B).

**Figure 6:**
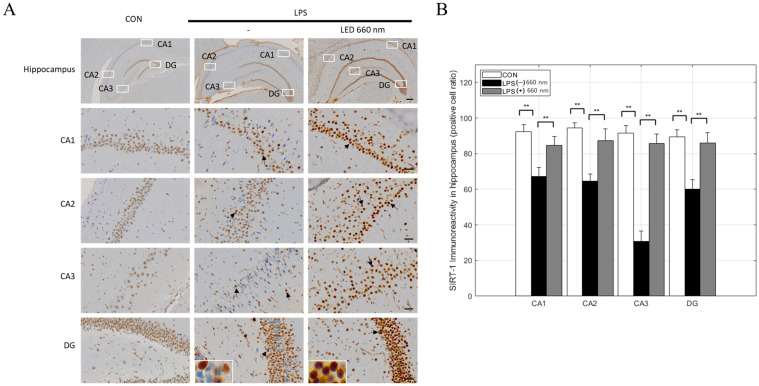
Light irradiation at 660 nm inhibits LPS-induced SIRT1 expression in the hippocampus. (**A**) Representative images of immunohistochemical staining for SIRT1 in the hippocampus (arrows). (**B**) SIRT1-positive cell numbers in the CA1, CA2, CA3, and DG regions (scale bars = 300 µm and 30 µm; ** *p* < 0.001).

SIRT1 is a major regulator of the cellular inflammatory response, inhibiting NF-κB activity and reducing cyclooxygenase-2 and inducible nitric oxide synthase production, thereby suppressing inflammation and increasing antioxidant gene expression. Compared to the CON group, the number of SIRT1-positive cells decreased significantly in all hippocampal areas in the LPS-treated group, especially CA3. In contrast, after phototherapy, SIRT1 expression increased to normal levels (Figure 6A,B).

## 4. Discussion

This study confirmed that PBMT using a 660 nm wavelength LED effectively mitigated the hippocampal damage and inflammatory response induced by LPS in rat brain tissue. Our findings suggested that PBMT exerts neuroprotective effects by inhibiting neuronal apoptosis and alleviates the inflammatory response by regulating the expression of inflammatory cytokines. In particular, increased SIRT1 expression was associated with enhanced neuroregenerative and neuroprotective functions. Therefore, PBMT is a promising new treatment modality against brain inflammation that can ameliorate the associated pathological changes through various neuroprotective mechanisms.

The morphological analysis of hippocampal areas CA1, CA2, and CA3 revealed that 660 nm phototherapy effectively maintained the structural integrity of pyramidal cells, reducing the lymphocyte infiltration, nuclear condensation, and cytoplasmic vacuolation induced by LPS. These results suggested that phototherapy protects neural structures against neuroinflammation. Notably, the decrease in GFAP expression in the CA3 and DG areas, TNF-α expression in the CA2 and DG areas, and IL-1β expression across the hippocampus further support the potential of 660 nm phototherapy for suppressing inflammatory responses and promoting neural function in neuroinflammation-related diseases. Similarly, various studies have shown that 660 nm phototherapy inhibited inflammatory cytokine expression and neuronal apoptosis in LPS-induced neuroinflammation models, restoring neurotransmitter and synaptic plasticity and cognitive function [24,27]. The current and previous findings highlight the potential of PBMT as a non-invasive approach in AD treatment.

Drugs such as galantamine [28], 4R-cembranoid [29], and meclizine [30] have shown similar effects in LPS-induced neuroinflammation models. Galantamine promoted the recovery of synaptic function and inhibited the production of inflammatory cytokines, such as IL-6, IL-1β, and TNF-α, along with NF-κB p65 signaling, thereby mitigating neuroinflammation and improving cognitive function. Resveratrol prevented cognitive impairment and hippocampal inflammatory response induced by chronic neuroinflammation in a mouse model [31]. These compounds have demonstrated potential for enhancing synaptic plasticity, reducing glial cell activation, inducing anti-inflammatory effects, and reducing GFAP, IL-1β, and TNF-α levels, offering various modalities for treating neuroinflammation. Similarly, we showed that PBMT induced potent neuroprotective effects through the suppression of neuroinflammation. However, unlike these drugs, this non-invasive procedure can likely be utilized with less side effects in brain inflammatory diseases, such as AD, PD, ALS, and MS, which may promote patient compliance.

PBMT effectively improves microvascular circulation in the brain [29], reduces oxidative stress [16], and suppresses inflammatory responses [32]. Not only does it inhibit neuroinflammation, but it also promotes overall brain health by enhancing neuroplasticity, neural regeneration, and cognitive function. This multidimensional outcome emphasizes its potential for mitigating the pathological changes associated with neuroinflammation and provides important foundational data for exploring its clinical application. Furthermore, combining PBMT with other treatment modalities may optimize the therapeutic effects, representing a new treatment paradigm.

This study has some limitations. While we described the potential therapeutic effects of PBMT in neuroinflammation, our use of a rat model limits the translatability of our findings to humans. In contrast to the human skull, the thinness of the skull in small animals allows for high light transmittance. Developing brain-insertable devices involves various challenges and necessitates further research. Additional studies are also required to evaluate the long-term effects and safety of PBMT and should explore methods to mitigate pathological changes more effectively through a multi-stimulus approach.

Owing to their potential side effects, conventional drug replacements are continually being developed. This study applied external stimulation using a 660 nm wavelength LED (Woori Tech. Daegu, Republic of Korea) for brain inflammation treatment. The development of implantable electronic medicine is expected to further advance the treatment of various diseases. However, the efficacy of bioelectronic treatments relies heavily on the effects of the underlying drugs. This study suggests that incorporating highly controlled irradiation systems in drug-based treatments may overcome various side effects, providing a promising framework for the development of bioelectronic medicines.

## 5. Conclusions

This study demonstrated that PBMT using a 660 nm wavelength LED effectively improved hippocampal damage and inflammatory response in a rat model induced by LPS. PBMT inhibited LPS-induced neuronal apoptosis and decreased GFAP, TNF-α, and IL-1β expression, thereby exerting neuroprotective effects and alleviating the inflammatory response. Specifically, the increase in SIRT1 expression was associated with enhanced neuroregenerative and neuroprotective functions. PBMT represents an innovative non-invasive treatment method for brain inflammation, specifically by establishing a protective local environment for hippocampal neurons that suppresses inflammatory responses. This study provides an important reference for exploring the clinical potential of PBMT, representing a new treatment paradigm for brain diseases. Barring further optimization and investigations into the efficacy of combinatorial treatments, PBMT may be established as a new standard for brain disease treatment.

## Figures and Tables

**Table 1:** Irradiation parameters.

Parameter [Unit]	Value
Center wavelength [nm]	660
Output mode	Continuous
Average radiant power [mW]	200
Spot area [cm^2^]	0.12
Irradiance at aperture [mW/cm^2^]	43
Frequency (kHz)	32
Beam profile	Round
Beam divergence [°]	120

## Data Availability

Data are contained within the article.
